# Optimization and purification of mannanase produced by an alkaliphilic-thermotolerant *Bacillus cereus* N1 isolated from Bani Salama Lake in Wadi El-Natron

**DOI:** 10.1080/13102818.2014.995932

**Published:** 2015-01-13

**Authors:** Ebaa Ebrahim El-Sharouny, Nabil M.K. El-Toukhy, Nermeen Ahmed El-Sersy, Abeer Abd El-Aal El-Gayar

**Affiliations:** ^a^ Botany and Microbiology Department, Faculty of Science, Alexandria University, Alexandria, Egypt; ^b^ Protein Research Department, Mubarak City for Scientific Research and Technology Applications, Alexandria, Egypt; ^c^ National Institute of Oceanography and Fisheries, Microbiology Laboratory Environmental Division, Alexandria, Egypt

**Keywords:** alkaliphilic-thermotolerant, *Bacillus cereus*, mannanase, optimization, purification

## Abstract

An alkaliphilic-thermotolerant *Bacillus cereus* N1 isolated from Bani Salama Lake, Wadi El-Natron, Egypt, was proved to produce mannanase enzyme. Optimization of the fermentation medium components using Plackett–Burman design was applied. Glucose and inoculum size were found to be the most significant factors enhancing the production of the enzyme. On applying optimized medium in the fermentation process, an enzyme productivity of 42.2 UmL^−1^ was achieved with 6.4 fold increase compared to the basal one. Mannanase was also extracted and purified using chromatography such as ion-exchange chromatographic and gel filtration methods. It was indicated that, the mannanase activity extracted and purified from the isolate *B. cereus* N1 was reduced to 321.6 U (about 36% of the whole mannanase in the culture filtrate) in comparison with the initial mannanase activity (900 U) and the total protein content reduced to 52 mg (the initial total protein content was 220 mg). However, the specific activity for the mannanase from *B. cereus* N1 at the end of the purification steps was found to be about 628 Umg^−1^ compared to 4.2 Umg^−1^ at the initial culture filtrate. It was also indicated that the mannanase enzyme was purified almost 149-fold.

## Introduction

Alkaliphiles thermophilic–thermotolerants are reported to be a rich source of alkaline thermotolerant active enzymes, that have numerous applications in many industrial processes due to an interest in their physiological adaptation to high pH and temperature.[1–3]

Of the enzymes now available for the industry, enzymes such as proteases, cellulases, lipases, mannanases and pullulanases are by far the most widely employed and they still remain the target biomolecules.[2] Many of the alkaliphilic *Bacillus* spp. were proven to be one of the main producers of those enzymes.[4,5]

Mannanase participates in the degradation of hemicellulose and similar polysaccharides by hydrolyzing the β-1, 4-glycosidic linkages within the main chain. The hemicelluloses are the second richest renewable energy substances on Earth. Mannan, glucomannan, galactomannan and galactoglucomannan are the major polysaccharides that constitute hemicelluloses.[6] β-1,4-Mannanases produced through biotechnology have become ubiquitous in industrial applications. They are widely applied in drug, printing and dyeing, textile, oil exploitation, production of animal feed, food industry, instant coffee processing, paper and biological research.[7] Furthermore, they are employed for the preparation of mannooligosaccharides used as non-nutritional food additives for selective growth of human beneficial intestinal microflora (bifidobacteria and lactobacilli). Moreover, they have shown to be effective in laundry detergents.[8] Most of these applications of β-mannanase can only be completed under extreme conditions.

Unlike conventional optimization, statistical optimization methods present a more balanced alternative to the OVAT (one-variable-at-a-time) approach, since it takes into account the interaction of variables in generating the process response.[9] Statistical experimental designs have been used for many decades and can be adopted on several steps of an optimization strategy, such as for screening experiments or searching for the optimal conditions of a targeted response.[9] Recently, the results analyzed by a statistical planned experiment are better acknowledged than those carried out by the traditional OVAT method. Some of the popular choices, applying statistical designs to bioprocessing, include the Plackett–Burman design.[10]

In this work, we report the optimization, purification and characterization of β-mannanse from an alkaliphilic-thermotolerant *Bacillus cereus* N1 isolated from Bani Salama Lake in Wadi El-Natron.

## Materials and methods

### Bacterial strains

*Bacillus cereus* N1 used in this study is an alkaliphilic-thermotolerant bacterium isolated from Bani Salama Lake, Wadi El-Natron, Egypt, previously identified by El-Sharouny et al. [11] using 16S rDNA with accession number KF164288.

### Media

Luria Broth medium (LB) [12] was used for maintenance of bacteria and seed culture. pH was adjusted at 10 ± 0.2.

Horikoshi-I medium [13] was used for fermentation experiments. pH was adjusted at 10 ± 0.2.

### Culture condition

Fermentation was carried out in 250 mL Erlenmeyer flask containing 100 mL of the Horikoshi-I media, inoculated with one mL inoculum of seed culture in LB medium and incubated at 37 °C, pH 10 and 180 rpm for 24 h.[4,14]

### Application of Plackett–Burman design for optimization of mannanase production

Application of a complete factorial design would require 2*^n^* experiments if *n* factors have to be investigated. In the present case, seven variables would lead to 128 trials, which is a very large number. Using a fraction of the factorial design without losing information about the main effects of variables can reduce the number of experiments.[15]

The Plackett–Burman experimental design, a fractional factorial design,[16] was used in this research to reflect the relative importance of various growth media component factors on mannanase activity in liquid cultures. In mannanase assay experiment, seven independent variables were screened in eight combinations organized according to the Plackett–Burman design matrix (Tables 1 and 2). For each variable, the high (+) and low (−) levels were tested. Medium components are given in gL^−1^ and inoculum size was added in ml with culture (A_550_ = 1.0).

**Table 1.** Factors examined as independent variables affecting mannanase enzyme activity and their levels in the Plackett–Burman experimental design.

		Levels		
Factors	Symbols	Low level (−1)	Basal medium (0)	High level (+1)
MgSO_4_.7H_2_O	Mg	0.1	0.2	0.5
K_2_HPO_4_	K_2_	0.5	1	1.5
NaCl	Na	5	10	15
Yeast extract	YE	2	5	7
Peptone	P	2	5	7
Inoculum size	IS	0.5	1	1.5
Glucose	G	5	10	15

**Table 2.** The Plackett–Burman experimental design matrix for seven factors.

Trials	Mg	K_2_	Na	YE	P	IS	G	Mannanase activity (UmL^−1^)	Mannanase specific activity Umg^−1^ protein
1	−1	−1	−1	+1	+1	+1	−1	7	6
2	+1	−1	−1	−1	−1	+1	+1	32	29.5
3	−1	+1	−1	−1	+1	−1	+1	22	29
4	+1	+1	−1	+1	−1	−1	−1	8.8	15.7
5	−1	−1	+1	+1	−1	−1	+1	15	13
6	+1	−1	+1	−1	+1	−1	−1	0	0
7	−1	+1	+1	−1	−1	+1	−1	10	14
8	+1	+1	+1	+1	+1	+1	+1	29	25
9	0	0	0	0	0	0	0	6.5	10.5

All trials were performed in duplicates and the average of mannanase activity results were treated as the responses. The main effect of each variable was determined by the following equation:

$$E_{xi}=(\Sigma M_{i+}-\Sigma M_{i-})/N,$$

where *E_xi_* is the variable main effect, *M_i_*_+_ and *M_i_*_−_ are mannanase production in trials where the independent variable (*xi*) was present in high and low concentrations, respectively, and *N* is the number of trials divided by 2.

A main effect with a positive sign indicates that the high concentration of this variable is nearer to optimum and a negative sign indicates that the low concentration of this variable is nearer to optimum. Using Microsoft Excel, statistical *t*-values for equal unpaired samples were calculated for determination of variable significance.

## Analytical methods

### Determination of mannanase activity

Mannanase activity was assayed in the culture supernatant after cell removal at 50 °C and pH 10, by measuring the reducing sugars liberated during the hydrolysis of galactomannan where the developed red brown colour was measured spectrophotometrically at 575 nm.[14,17] One unit of activity was defined as the amount of enzyme catalyzing the production of 1 μmol of the reducing sugar per minute, using mannose as the standard.

### Estimation of total protein content

The total protein content of the samples was determined according to Lowry et al. [18].

### Purification of mannanase enzyme

The bacterium was grown in a batch culture to obtain the crude enzyme for purification. All purification steps were carried out at 4 °C, unless otherwise stated. Ion-exchange chromatographic methods and gel filtration methods were used to get a purified mannanase from bacterial strains.

### Separation using anion-exchange column chromatography

 The diethylaminoethanol (DEAE)-sepharose CL6B as an example for anion exchange column chromatography [19] was used to purify the mannanase enzyme from the isolate *B. cereus* N1. The column equilibrated with 20 mmol/L tris-acetate buffer, pH 7, and the suspension was filled into a column (3.5 × 20 cm). The column was washed with 300 mL of equilibration buffer and then the bound proteins were eluted with a linear gradient from 0 to 1.0 mol/L NaCl (100 mL) in 20 mmol/L tris-acetate buffer, pH 7. The flow rate used was 1 mL min^−1^ and fractions collected were examined for total protein content and for mannanase activity. The fractions which contained the highest mannanase activity were pooled and dialyzed twice against 5 L of 20 mmol/L tris-acetate buffer, pH 7.

### Separation using gel filtration column chromatography

Gel filtration column chromatographic methods were used as one of the steps to discard the foreign proteins depending on the molecular mass principles and purify the mannanase enzymes in polishing form from *B. cereus* N1. This separation was carried out through fast protein liquid chromatography.

### Separation using the 32/60 Sephacryl S-100 high resolution (HR) column chromatography

Gel filtration, as final step of purification for mannanase enzyme, was conducted on a 2.5 × 60 cm column pre-packed with Sephacryl S-100 HR. The column was equilibrated with 50 mmol/L sodium acetate buffer, pH 6, containing 0.2 mol/L NaCl. The elution rate was 60 mLh^−1^.

All the fractions (30 fractions, 4 mL each) collected were tested for total protein content and for mannanase activity. Fractions containing mannanase activities were pooled and dialyzed twice against 5 L of 20 mmol/L sodium acetate buffer, pH 6.

### Concentrating the purified protein

The purified mannanase was concentrated using the ultrafiltration tubes at a speed of 5000 rpm for 30 min at 4 °C in Centricon 10 (Amicon, USA) ultrafiltration concentrators (membrane cut-off of 10 kDa). The used ultrafiltration tubes were cleaned by centrifuging first with 2 mL bidistilled water to ensure the cleanliness of the ultrafiltration membrane tubes.

The samples were collected from the ultrafiltration-membrane tubes to other small tubes by attaching the tubes to each other and reverse the position of the ultafiltration-membrane tubes in the centrifuge and starting centrifugation at a speed of 1500 rpm for 15 min to avoid crashing of the ultrafiltration membrane.

### Polyacrylamide gel electrophoresis (PAGE)

The denaturing SDS-PAGE (SDS, sodium dodecyl sulfate) has been used for detecting the mannanase enzyme homogeneity and estimation of its molecular mass using protein marker. The gels were prepared according to the method of Laemmli [20] with some modifications using 10% polyacrylamide gels and in the help of using a molecular mass marker containing wide range of proteins ranging from 14.2 to 205 kDa.

## Results and discussion

The thermoalkaliphiles and alkaliphiles are promising in terms of production of biomolecules suited for industrial applications.[5] Alkaliphilic *Bacillus* species are the most characterized organisms among alkaliphiles. They produce so many extracellular alkaline-adapted enzymes that they are often good sources for industrial enzymes.[1] Vijayalaxmi et al. [21] reported the production of alkaline β-mannanase by alkaliphilic *Bacillus* sp. N16-5 isolated previously from sediment of Wudunur Soda Lake in Inner Mongolia, China.

### Screening of significant variables affecting the production of mannanase enzyme from *Bacillus cereus* N1 using Plackett–Burman design

Plackett–Burman design, an efficient technique for medium component optimization,[22] was employed to identify significant variables that enhance mannanase production and to find out their probable optimal levels in a limited number of experiments. In this study, seven variables were analyzed with regard to their effects on enzyme production using a Plackett–Burman design.(Table 1)

The selected variables included nutritional factors such as carbon source (glucose), nitrogen source (yeast extract and peptone), source of phosphates (K_2_HPO_4_), other complementary salts (MgSO_4_.7H_2_O and NaCl) and finally, inoculum size. Environmental factors were kept constant with basal values of pH 10 and culture volume of 100 mL. The seven variables were evaluated by eight experiments and the levels of each variable were determined based on prior experience with the system. The independent variables were examined and their settings are shown in Table 1.

**Figure 1.**
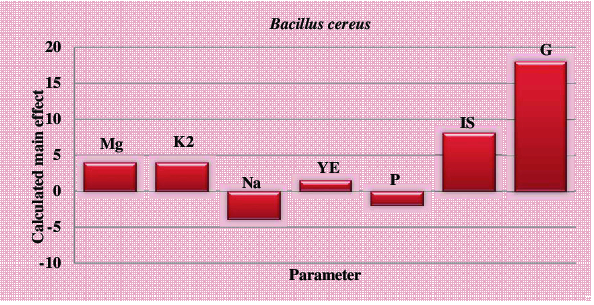
Elucidation of cultivation factors affecting mannanase production by *B. cereus* N1 using Plackett–Burman experimental design.

The responses in Table 2 show a wide variation in mannanase activity, ranging from 0 to 32 UmL^−1^ corresponding to the combined effect of the seven parameters in their specific ranges. For determination of variable significance, statistical *t*-values for equal unpaired samples were calculated with respect to observations. The necessary statistical analyses of this experiment are shown in Table 3. The main effect of each variable upon mannanase was estimated and presented graphically in Figure 1.

**Table 3.** Statistical analysis of the Plackett–Burman experimental result for *B. cereus* N1.

	Mannanase activity			
Factors	Main effect	*t*-value	*P*-value	Significance level
MgSO_4_.7H_2_O	3.95	0.468	0.66	–
K_2_HPO_4_	3.95	0.468	0.65	–
NaCl	−3.95	−0.468	0.65	–
Yeast extract	1.45	0.16	0.87	–
Peptone	−1.95	−0.228	0.83	–
Inoculum size	8.05	1.01	0.35	–
Glucose	18.05	4.1	0.0093	99%

**Figure 2.**
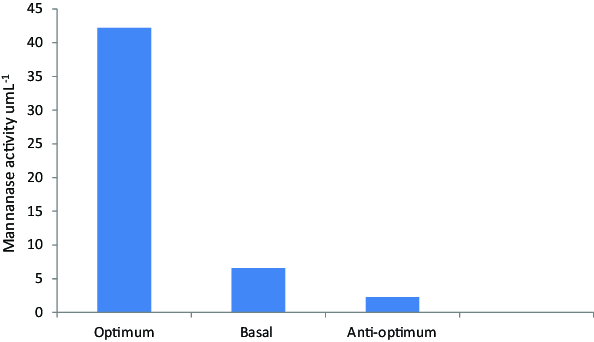
Verification experiment for mannanase activity produced by *B. cereus* N1.

As shown in Figure 1, the main effect results of strain *B. cereus* N1 point out that high level of MgSO_4_.7H_2_O, K_2_HPO_4_, inoculum size and glucose in the growth medium affects mannanase activity positively. This figure also suggests that low concentration levels of NaCl, yeast extract and peptone would result in high mannanase activity. Therefore, one could state that the enzyme production by *B. cereus* N1 was mainly dependent on glucose and NaCl, i.e. the high concentration of NaCl had the most significant negative effect on mannanase production, while the high concentration of glucose had the most significant positive effect on mannanase production. Therefore, decreasing NaCl concentration and increasing glucose concentration in the culture medium will enhance the extracellular mannanase production.

Lin et al. [4] used a 2^2^ factorial design, which was employed to optimize the medium compositions for the production of alkaline β-mannanase by alkaliphilic *Bacillus* sp. N16-5 isolated previously from sediment of Wudunur Soda Lake in Inner Mongolia, China. They found that the production of alkaline β-mannanase by strain N16-5 was induced by addition of some substrates containing β-mannan into the medium. The most suitable carbon source was locust bean gum and the nitrogen sources were peptone and yeast extract.

The significant variables were identified by statistical analysis of the Plackett–Burman experiment using the *t*-test supported by Excel Microsoft Office to determine the statistical significance of the measured response and calculated main effects for *Bacillus cereus* N1 (Table 3). Significance of coefficients has been reported to be directly proportional to *t*-test and inversely to *P*-value. The smaller the *P*-values, the bigger the significance of the corresponding coefficient.[23]

Some researchers employed confidence levels greater than 70% as significant effect levels.[24] Other investigators find that, confidence levels greater than 90% are acceptable [25] or even levels greater than 95% are considered to have a significant effect on the response.[26] However, an intermediate value of *P* = 0.2 corresponded to a statistical confidence of 80% was chosen in this study, and hence, any component showing a statistical confidence equal and/or higher than 80% was considered significant.

According to the data obtained from the Plackett–Burman experimental results and all calculations related to this experimental design, it can be predicted that high microbial mannanase production could be obtained using a medium formula of the following composition (gL^−1^): glucose, 15; yeast extract, 2; K_2_HPO_4_, 1.5; NaCl, 5; peptone, 7; inoculum size (mL),1.5 and MgSO_4_.7H_2_O, 0.5. The pH was constant at 10 and was adjusted by adding 2 mL of NaHCO_3_ after the sterilization process aseptically. Also galactomannan was added to the media at a constant weight of 4 gL^−1^.

### Verification experiment

In order to validate the obtained data and to evaluate the accuracy of the applied Plackett–Burman statistical design, a verification experiment was carried out in duplicates to predict the near optimum levels of independent variables. The data were examined and compared to the basal and anti-optimized medium in Figure 2, where one could notice that during the verification experiment applied on *B. cereus* N1, the mannanase production is raised 6.4 fold when growing in optimized medium.

The results of this experiment suggested that the most effective variables, concerning mannanase activity are the concentrations of glucose in addition to the inoculum size in mL. Among those, statistical analyses of the data (*t*-test) demonstrated the significance of glucose and inoculums size in mL.

Inoculum size has significant effect on the mannanase production. Therefore, a suitable inoculum size is needed to have the highest mannanse production as lower inoculum size was able to slow down the biomass proliferation. Thus, the degradation of the substrates by the microbes is slower and affects the metabolite production.[27]

On the other hand, regarding the effect of glucose concentration, Youssef et al. [28] studied the effect of carbon sources on the production of β-mannanase using the basal culture medium supplemented with 2% mannan as a control. In other trials, mannan was replaced by equal amounts of different carbon sources, as a sole carbon source. It was found that the lowest mannanase activity was obtained in cultures containing glucose as a carbon source, which was found to promote mannanase activity but in a much lower level rather than that achieved if replaced by coconut. However, still few investigators used glucose or any other readily metabolizable carbohydrates as a sole carbon source with significant production of mannanase.[29] Generally, the synthesis of mannanase enzyme is inducible in microbial cells and appears to be controlled by carbon repression when more easily metabolizable carbon sources, e.g. glucose, are present in the culture medium together with a substrate suitable for inducing enzyme synthesis. Hence, the enzyme formation in the organism starts only when the repressing glucose is completely metabolized, although moderate levels of enzymes were produced when glucose was used as the only carbon source.[29]

### Extraction and purification of mannanase enzyme

A scheme of extracted and purified mannanases enzyme for *B. cereus* N1 was shown in Table 4. It was indicated that the mannanase activity extracted and purified from the isolate *B. cereus* N1 was reduced to 321.6 U (about 36% of the whole mannanase in the culture filtrate) in comparison with the initial mannanase activity (900 U) and the total protein content was reduced to 52 mg in comparison with the initial total protein content of 220 mg. However, the specific activity for the enzyme at the end of the purification steps was found to be about 628 Umg^−1^ comparing to 4.2 Umg^−1^ at the initial culture filtrate. It was also indicated that at the end of the extraction steps, the mannanase enzyme was purified almost 149-fold.

**Table 4.** Purification scheme of mannanase enzyme from the isolate *B. cereus* N1.

Purification step	Volume (mL)	Mannanase activity (UmL^−1^)	Total protein (mgmL^−1^)	Specific activity (Umg^−1^)	Yield (%)	Purification (fold)
Culture filtrate	1000	0.9	0.22	4.22	100.00	1.00
Concentrating using ultrafiltration Amicon	100	8.8	1.88	4.68	97.78	1.11
DEAE-sepharose CL6B	10	60.2	0.71	84.79	88.89	20.09
32/60 Sephacryl S-100 HR	4	80.4	0.13	628.28	35.73	148.88

### Anion-exchange column chromatography

The DEAE-sepharose chromatography, as an anion exchange chromatography, was used in order to separate and purify the mannanase enzyme from the isolate *B. cereus* N1.

The concentrated suspension by the ultrafiltration Amicon system containing about 188 mg of total foreign proteins and 880 total units of mannanase activity was filled into the DEAE-sepharose column at pH 7. About 88.9% of the total mannanase enzyme (602 U) and about 7.1 mg of the total protein were recovered (Table 4). A 20-fold purification indicated that DEAE-sepharose CL 6B is considered to be a good step for separating and purifying the mannanase enzyme from the isolate *B. cereus* N1.

**Figure 3.**
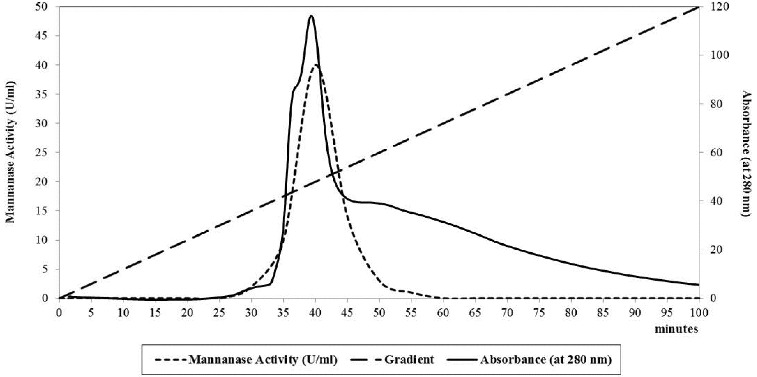
DEAE-sepharose CL6B column as anion exchange column chromatography of *B. cereus* N1 mannanase preparation. Buffer: 20 mmol/L tris-acetate, pH 7.

Figure 3 shows the elution diagram of the mannanase enzyme using DEAE-sepharose CL6B and it indicates a sharp peak of the mannanase enzyme elution, which gives the maximum activity after the gradient per cent reach about 45% from the sodium chloride.

### Gel filtration chromatography

Gel filtration technique was used as a final step for mannanase purification. Chromatographic columns, such as 32/60 Sephacryl S-100 HR, were used in order to purify the mannanase enzyme in the final form.

### 32/60 Sephacryl S-100 HR chromatography

The dialyzed sample from the isolate *B. cereus* N1, which contains 7.1 mg of total proteins and 602 total units of mannanase activity, was filled into the 32/60 Sephacryl S-100 HR chromatographic column at pH value of 6.0.

**Figure 4.**
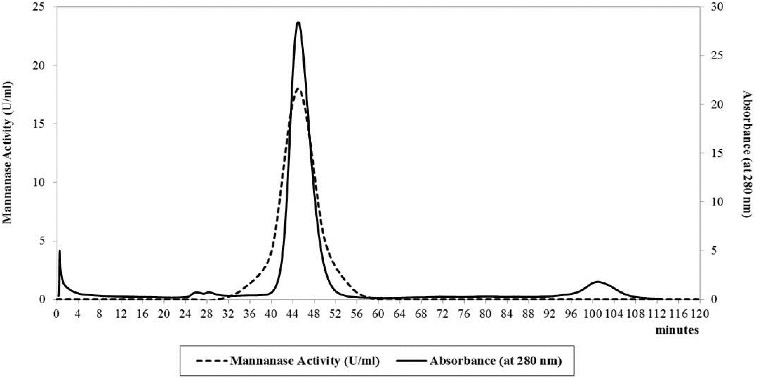
Sephacryl S-100 HR column as a gel filtration chromatography of *B. cereus* N1 mannanase preparation. Buffer: 50 mmol/L sodium acetate containing 0.2 mol/L NaCl, pH 6.

The pooled collected fractions showed a recovery of about 35.7% of the total mannanase enzyme (321.6 U) and only 0.52 mg of the total protein (Table 4). About 149-fold purification showed the importance of using gel filtration as a method of separation and purifying the mannanase enzyme. Figure 4 illustrates the elution diagram of the mannanase enzyme using 32/60 Sephacryl S-100 HR chromatographic column and it indicates a sharp peak of the mannanase enzyme.

Chandra et al. [30], who isolated and purified mannanase enzyme from *Paenibacillus cookii* and *Bacillus* sp., stated that about 6.4% and 17.2% mannanase were recovered, and 90.2- and 48.5-fold of purification were achieved at this stage. Moreover, mannanase from alkaliphilic *Bacillus* sp. N16-5 has been investigated by Ma et al. [14]. The enzyme was purified with a yield of 8.8%. An approximately 15.8-fold purification to a specific activity of 5065 Umg^−1^ of protein was obtained for the mannanase activity when measured in 50 mM glycine–NaOH buffer (pH 9.5).

### Testing of the enzyme purity and estimation of their molecular mass

In order to prove the purity of the mannanse enzyme extracted and purified from *B. cereus* N1, SDS gel electrophoresis was used. Using SDS gel electrophoresis is considered to be an indicator not only for of the purity of the mannanase enzyme, but also as a method for detecting the molecular mass of the purified enzymes.

**Figure 5.**
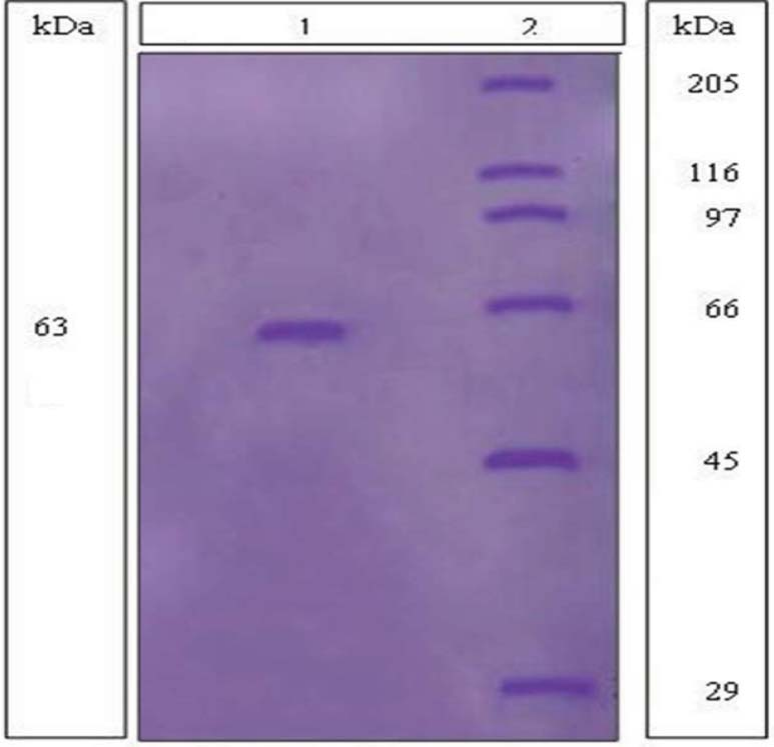
SDS-gel polyacrylamide electophoresis (PAGE), showing the purified mannanase from *B. cereus* N1. Lane 1: Showed mannanase extracted and purified from *B. cereus* N1. Lane 2: Showed the SDS-6H standard proteins (kDa).

It showed on the gel in our case that only a single band appeared for mannanase from *B. cereus* N1 and no other bands appeared. This indicated the purity of the isolated mannanase enzyme with molecular mass estimated by comparing with the marker to be about 63 kDa (Figure 5). This result is similar to that found in the literature for mannanase from other separate sources.[31,32]

## Conclusion

In this work, an alkaliphilic-thermotolerant *Bacillus cereus* N1 was isolated from Bani Salama Lake, which is one of the famous soda lakes located in Wadi El-Natron, Egypt, that has also been rarely investigated for its microflora. Optimization of the fermentation medium components of the isolate was applied, using Plackett–Burman design, leading to 6.4 fold increase in enzyme activity. Mannanase was also extracted and purified using chromatography such as ion-exchange chromatographic and gel filtration methods, where the specific activity for the enzyme reached about 628 Umg^−1^ compared to 4.2 Umg^−1^ at the initial culture filtrate. It was also indicated that the mannanase enzyme at the end of the extraction steps was purified almost 149-fold and has a molecular mass of 63 kDa.
